# The Compulsory Notification of Tuberculosis

**Published:** 1906-07-21

**Authors:** 


### The Hospital

A Journal of

The Medical Sciences and Hospital Administration.

Vol. XL.—No. 1,035. SATURDAY, JULY 21, 1906.

### The Compulsory Notification of Tuberculosis.

An increased appreciation of the economic and social relationships of tuberculosis has been one of the necessary results of the recognition of a specific micro-organism as the essential causes of the disease. Prior to that event the public mind was disposed, with more or less resignation, to accept tuberculosis as one of the mysterious decrees of Providence by which families and generations are handed over as victims to an inevitable fate. But with a recognition of the true etiology of the disease, the point of view has been completely changed. Gradually the significance of this new truth has become apparent, and the question is now being asked from various quarters whether, as the evil arises from without and not from within, measures cannot be framed to check or curtail its deadly prevalence. This question is urged on those charged with the care of the health of the community with all the greater emphasis from the fact that the public recognises that other diseases due to specific germs have been brought under efficient control. It seems a readily justified position to claim that if typhus fever can be practically abolished, tuberculosis, which has an analogous causation, ought to yield to a similar discipline.

Now it must be freely recognised that the compulsory notification of certain diseases as "infectious" has been a large and successful influence in protecting the public from outbreaks of these diseases. The Act of Parliament under which such notification is enforced contains, as we think at least, serious defects; but it can claim the justification of success. Hence, at first sight, it appears a reasonable proposal to apply the same procedure to tuberculosis—that is, to pronounce this to be an "infectious" disease, and to demand that every case when recognised shall be formally notified to the public health authority. A very short consideration of the facts, however, is sufficient to show that such a demand is impracticable. In one form or another tuberculosis is one of the most widespread diseases. Hence, on the mere question of expense, a proposal to notify every instance of it must at once be recognised as impossible. From such a suggestion even the most extreme advocate of the doctrine of "infection" is compelled to shrink. The claim thus becomes contracted, and, indeed, except for a few disciples of a doctrine of "thorough," is hardly urged outside the area of phthisis pulmonalis. This, however, is roundly denounced as "infectious," and therefore as suitable for subjection to measures of sanitary police.

It is fair to say that the whole case of the advocate of compulsory notification of phthisis pulmonalis rests upon the contention that the disease is "infectious." Unless this is the case there is no adequate ground for interference. Now whilst no one will deny that in certain conditions and circumstances the disease can be conveyed from one person to another, it is not true that pulmonary phthisis is infectious in the sense in which this term can be applied to, say, typhus fever, scarlet fever, or small-pox. In these the risk from exposure is extremely high, and is not sensibly diminished by any conditions which can be readily commanded. In phthisis pulmonalis, on the other hand, the risk is very slight, and by attention to easily observed precautions is practically eliminated. Therefore it is hardly fair to declare to the public that tuberculosis is an "infectious" disease, for the interpretation put upon this statement will be that all tuberculous patients are a danger to the community. By all means let the truth be known; but the truth is that whilst tuberculosis is in a limited sense communicable from person to person, such communication only occurs under special and peculiar conditions. Chief among these are sleeping with a consumptive person or living with such a person in ill-ventilated apartments. It is a commonplace, too, that it is through the sputa that the disease is spread, and consequently measures ought always to be taken to disinfect or to destroy this. But granted due attention to these conditions, it may be safely contended that the victim of phthisis pulmonalis is no source of danger to his friends and neighbours, and that he is therefore *not* the subject of an "infectious" disease.

If the argument above stated is sound, whilst there may be ample warrant for education of the patient, there is surely none for publicly attaching to him a label which cannot fail to make him a marked member of the community, and which will certainly interfere with his social happiness and his business success. Unless there is distinct and definite danger we must hold that the community has no right so seriously to penalise the individual citizen. The disabilities attending notification are serious in themselves, and it must be remembered that in the majority of cases they would extend over a considerable term of years. In advanced cases of the disease, and in circumstances where proper protection for others cannot be secured, it may be well to promote public intervention; but this is a very different claim from the proposal that every case of pulmonary tubercle, irrespective of degree, surroundings, or other circumstances, shall be compulsorily notified to the local public health authority.

